# Breast cancer bone metastases are attenuated in a Tgif1-deficient bone microenvironment

**DOI:** 10.1186/s13058-020-01269-8

**Published:** 2020-04-09

**Authors:** Marie-Therese Haider, Hiroaki Saito, Jennifer Zarrer, Kevin Uzhunnumpuram, Sankari Nagarajan, Vijayalakshmi Kari, Michael Horn-Glander, Stefan Werner, Eric Hesse, Hanna Taipaleenmäki

**Affiliations:** 1 Molecular Skeletal Biology Laboratory, Department of Trauma, Hand and Reconstructive Surgery, University Medical Center Hamburg-Eppendorf, Hamburg, Germany; 2 Institute of Molecular Musculoskeletal Research, University Hospital, LMU Munich, Munich, Germany; 3 Department of General, Visceral and Pediatric Surgery, University Medical Center Göttingen, Göttingen Center for Molecular Biosciences, Göttingen, Germany; 4 Present address: Cancer Research UK Cambridge Institute, University of Cambridge, Cambridge, UK; 5 University Cancer Center Hamburg, University Medical Center Hamburg-Eppendorf, Hamburg, Germany; 6 Institute of Tumor Biology, University Medical Center Hamburg-Eppendorf, Hamburg, Germany

**Keywords:** Breast cancer, Bone metastases, Osteoblasts, Tgif1

## Abstract

**Background:**

Osteoclast activation is a hallmark of breast cancer-induced bone disease while little is known about the role of osteoblasts in this process. Recently, we identified the homeodomain protein TG-interacting factor-1 (Tgif1) as a crucial regulator of osteoblast function. In this study, we demonstrate that lack of Tgif1 also restricts the progression of breast cancer bone metastases.

**Methods:**

Transwell migration assays were used to investigate the osteoblast-breast cancer cell interaction in vitro. Molecular analyses included RNA sequencing, immunoblotting, and qRT-PCR. To determine the role of Tgif1 in metastatic bone disease, 4T1 breast cancer cells were injected intracardially into mice with a germ line deletion of Tgif1 (*Tgif1*^*−/−*^) or control littermates (*Tgif1*^*+/+*^). Progression of bone metastases and alterations in the bone microenvironment were assessed using bioluminescence imaging, immunofluorescence staining, confocal microscopy, and histomorphometry.

**Results:**

Medium conditioned by osteoblasts stimulated breast cancer cell migration, indicating a potential role of osteoblasts during bone metastasis progression. Tgif1 expression was strongly increased in osteoblasts upon stimulation by breast cancer cells, demonstrating the implication of Tgif1 in the osteoblast-breast cancer cell interaction. Indeed, conditioned medium from osteoblasts of *Tgif1*^*−/−*^ mice failed to induce breast cancer cell migration compared to control, suggesting that Tgif1 in osteoblasts augments cancer cell motility. Semaphorin 3E (Sema3E), which is abundantly secreted by *Tgif1*^*−/−*^ osteoblasts, dose-dependently reduced breast cancer cell migration while silencing of Sema3E expression in *Tgif1*^*−/−*^ osteoblasts partially restored the impaired migration. In vivo, we observed a decreased number of breast cancer bone metastases in *Tgif1*^*−/−*^ mice compared to control littermates. Consistently, the presence of single breast cancer cells or micro-metastases in the tibiae was reduced in *Tgif1*^*−/−*^ mice. Breast cancer cells localized in close proximity to Endomucin-positive vascular cells as well as to osteoblasts. Although Tgif1 deficiency did not affect the bone marrow vasculature, the number and activity of osteoblasts were reduced compared to control. This suggests that the protective effect on bone metastases might be mediated by osteoblasts rather than by the bone marrow vasculature.

**Conclusion:**

We propose that the lack of Tgif1 in osteoblasts increases Sema3E expression and attenuates breast cancer cell migration as well as metastases formation.

## Background

Breast cancer is one of the most prevalent malignancies and constitutes a tremendous medical and socio-economic burden [1]. Increased awareness combined with advancements in screening methods and therapies has led to an improved prognosis and survival rate of breast cancer patients [2]. Nevertheless, after initial treatment, distant metastases frequently occur after years or even decades of a disease-free interval. Bone is the most frequent site for metastases in breast cancer, and more than 70% of patients with an advanced disease suffer from bone metastases [3, 4]. These patients have a high morbidity caused by skeletal-related events due to the predominantly osteolytic nature of the disease. Furthermore, the survival rate remains poor with only 25% of patients living more than 3 years upon diagnosis of breast cancer bone metastases [5].

Breast cancer-induced bone destruction is a consequence of a disturbed bone remodeling, a well-characterized process known as the “vicious cycle” of bone metastases [6]. Briefly, tumor-derived factors including parathyroid hormone-related protein (PTHrP) stimulate bone-forming osteoblasts to secrete receptor activator of nuclear factor kappa-B ligand (RANKL) as well as other cytokines. The increase in RANKL activates bone-resorbing osteoclasts and subsequent bone destruction. This results in the release of matrix-derived growth factors, such as transforming growth factor-β1 (TGF-β1), which further stimulate tumor growth [6, 7]. Although novel therapeutic options and targets are emerging (e.g., tyrosine kinase inhibitors, microRNAs [8–11]), the disease remains incurable once osteolytic lesions have developed.

Upon entering the bone, breast cancer cells are exposed to a heterogeneous bone microenvironment, which comprises different cell types and non-cellular cues including growth factors, cytokines, and the extracellular matrix [12]. Each component of the bone microenvironment has important roles in supporting tumor cell quiescence, metastases initiation, and progression as well as resistance and/or response to anti-cancer therapy [13–20]. Within the bone microenvironment, the endosteal (osteoblasts, osteoclasts, adipocytes) and the vascular (vascular endothelial cells, pericytes) niches regulate hematopoietic stem cell (HSC) renewal and differentiation via cytokines, intracellular signals, and cell-cell contacts (e.g., integrins, cadherins) [21–25]. It has been suggested that tumor cell homing to bone, quiescence, and metastatic growth are mediated via these niche-controlling signals [15, 17–20, 26–28]. Hence, modification of the bone microenvironment including the niches might offer novel therapeutic approaches to target metastatic bone disease. However, although the understanding of how tumor cells and the bone microenvironment influence each other increases continuously, the knowledge about early events of bone metastases, especially regarding the stimuli that initiate metastatic growth, is rather limited.

High abundance of the homeodomain protein TG-interacting factor 1 (Tgif1) has been associated with poor patient survival in various cancers, including upper urinary tract urothelial carcinoma, colorectal cancer, and breast cancer [29–31]. Besides its role in carcinogenesis, we recently identified Tgif1 as a novel target gene of the canonical Wnt and of the Parathyroid hormone receptor type 1 (PTH1R)-dependent signaling pathways in bone [32]. Furthermore, we determined its important role as a novel regulator of bone homeostasis by demonstrating that the deletion of Tgif1 results in a low bone turnover phenotype in vivo [32]. Given the crucial regulatory function of Tgif1 in both tumorigenesis and bone remodeling, we hypothesized that Tgif1 could also impact the initiation and progression of breast cancer bone metastases.

Here, we report that Tgif1 expression is strongly increased in osteoblasts upon stimulation by metastatic breast cancer cells, suggesting a potential role of Tgif1 in the osteoblast-breast cancer cell interaction. Furthermore, our results reveal that medium conditioned by osteoblasts stimulates breast cancer cell migration in a Tgif1-dependent manner. This indicates that Tgif1 in osteoblasts may also play a role during the early stages of bone metastasis. In vivo deletion of Tgif1 in mice attenuates the progression of bone metastases and protects from breast cancer-induced bone destruction. Together, our findings establish osteoblasts and Tgif1 as important regulators of breast cancer cells in the bone microenvironment and of the formation of breast cancer bone metastases.

## Methods

### Cell culture

Two sub-clones of the mouse mammary epithelial cell line 4T1, stably expressing green fluorescence protein (GFP) or GFP together with luciferase (luc), hereafter referred to 4T1-GFP and 4T1-GFP-luc were used. For the establishment of a stable luciferase expression in 4T1 cells (purchased from ATCC), the luc2 gene was cloned into the LeGO-iG2 plasmid (PMID: 18362927) using the EcoRI restriction site. To produce infectious particles, the second-generation lentiviral packaging system was used by transient transfection of 293T cells using Lipofectamine 2000. For transduction, the viral supernatant was added in a 1:10 dilution to 50% confluent recipient cultures. Positive selection of GFP-positive cells was performed 72 h after transduction by fluorescence-activated cell sorting (FACS). The 4T1-GFP cells were kindly provided by Dr. Sonja Loges. The 4T1 cells were cultured in RPMI 1640 medium (Gibco, 61870-010) supplemented with 10% fetal bovine serum (FBS, Gibco, 10270-106) and 1% penicillin-streptomycin (P/S, Gibco, 15070-063). The mouse osteoblast cell line MC3T3-E1 and the human breast cancer cell line MDA-MB-231 (both obtained from ATCC) were cultured in α-MEM (Gibco, 22571-020) supplemented with 10% FBS and 1% P/S.

For calvarial osteoblast cultures, the calvariae were dissected from 1- to 3-day-old mice and digested sequentially (4 × 25 min) in α-MEM containing 0.1% collagenase and 0.2% dispase (both from Roche). The first cell fraction was discarded; fractions 2 to 4 were collected, combined, and expanded in α-MEM containing 10% FBS and 1% P/S.

To obtain conditioned medium, cells were washed twice with phosphate-buffered saline (PBS) and serum starved for 24 h in α-MEM supplemented with 1% FBS and 1% P/S. On the next day, the medium was collected, centrifuged for 5 min at 900*g*, and stored at − 80 °C. For all experiments, 50% medium conditioned by cancer cells (CCM) or medium conditioned by osteoblasts (ObCM) was used (diluted in α-MEM + 1% FBS + 1% P/S). MC3T3-E1 cells were stimulated with CCM (from 4T1 or MDA-MB-231 cells) for the indicated periods of time. Treatment of osteoblasts with a selective ERK1/2 inhibitor (SCH772984, Santa Cruz Biotechnology) was performed prior to the stimulation with CCM.

### Transwell migration assay

For transwell migration assays (BD Biosciences, BIOCOAT® Cell culture inserts, 354578), 2 × 10^4^ breast cancer cells per well were allowed to migrate through 8-μm pores towards control medium (α-MEM + 1% FBS + 1% P/S, referred to as Ctrl), ObCM, or towards α-MEM supplemented with recombinant Semaphorin 3E (Sema3E, R&D Systems, 100-500 ng/ml) for 6 h. Migrated cells were stained using Giemsa’s azur eosin methylene blue solution (Merck, HX8389304). Cells within 4 fields of view of interest were counted using the OsteoMeasure system (Osteometrics) using a × 10 objective (Olympus UPlan Fl 10x/0.30 ∞/).

### RNA sequencing

For RNA sequencing, osteoblasts were isolated from the calvariae of *Tgif1*^*−/−*^ mice and *Tgif1*^*+/+*^ control littermates as described above. Libraries were prepared from 1 μg total RNA using the NEBNext Ultra RNA Library Preparation Kit for Illumina (NEB). The size of the library was measured using a Bioanalyzer 2100 (Agilent Technologies), and a 51-bp single-end sequencing was used for RNA sequencing. After aligning the reads using Bowtie2 with mm9 cDNA transcriptome, reads were counted with a custom ruby script and DESeq was applied to identify differentially expressed genes.

### Mouse model of bone metastasis

To determine the role of Tgif1 during the establishment and progression of breast cancer bone metastases, 8–10-week-old female mice with a germ-line deletion of Tgif1 (*Tgif1*^*−/−*^) or control littermates (*Tgif1*^*+/+*^) were used [33]. Mice were backcrossed from C57Bl/6 background to BALB/c background for at least ten generations. Mice were injected intracardially with 1 × 10^5^ 4T1 breast cancer cells (4T1-GFP or 4T1-GFP-luc) and sacrificed 5, 7, and 9 days after tumor cell injection. Metastasis formation was monitored on day 7 using bioluminescence imaging (BLI) and quantified using the Living Image Software. To quantify the number of metastases formed, BLI signals were counted per leg for each mouse. For dynamic histomorphometry, 8-week-old tumor-free mice were injected intraperitoneally 7 and 2 days prior to the sacrifice with calcein (Sigma, C0875, 20 mg/kg) and demeclocycline (Sigma, D6140, 20 mg/kg), respectively.

### Sample preparation

For paraffin embedding, bones were fixed in 4% paraformaldehyde (PFA, pH 7.4, in PBS) for 48 h at 4 °C, followed by decalcification in 0.5 M EDTA/0.5% PFA for 14 days. Decalcified bones were cut into 5-μm-thick sections. For embedding in methylmethacrylate (MMA), bones were fixed in 4% PFA for 48 h. Fixed, non-decalcified bones were embedded in MMA and cut into 4-μm-thick sections.

### Immunofluorescence staining and imaging

To visualize the bone marrow vasculature and single tumor cells by immunofluorescence, long bones were fixed in 4% PFA for 4 h at 4 °C and decalcified in 0.5 M EDTA (pH 8, in PBS) for at least 24 h. Bones were then embedded in gelatin [34–36]. Samples were stored at − 80 °C prior to cutting into 30-μm-thick sections. Immunofluorescence staining of the vascular endothelial cell marker Endomucin (1:100, Endomucin V.7C7, rat monoclonal, Santa Cruz, sc-65495) and of the osteoblast marker Osterix (1:200, Osterix antibody (A-13), rabbit polyclonal, Santa Cruz, sc-22536) was performed on bone sections [34–36] (see Table 1 for antibody details). The GFP signal of the 4T1-GFP cells was retained during gelatin embedding, allowing the visualization of the immunofluorescence without prior staining. Images were acquired using the Leica SP5 confocal microscope, × 20 objective (20x HC PL APO CS IMM/CORR, NA: 0.70, WD (mm): 0.59 (W), =.17 (oil)). The presence and localization of 4T1-GFP breast cancer cells in long bones were determined using confocal microscopy 5 days after injection. For each mouse, 3–4 non-serial sections were analyzed.

**Table 1:** Antibodies used in this study

Antibody	Supplier	Concentration
Endomucin V.7C7, rat monoclonal	Santa Cruz, sc-65495	1:100
Osterix A-13 rabbit polyclonal	Santa Cruz, sc-22536	1:200
Alexa Fluor 546 goat-anti rat	Life Technologies, A11081	1:400
Alexa Fluor 546 donkey-anti rabbit	Life Technologies, A10040	1:400
Tgif1, rabbit monoclonal	Abcam, ab52955	1:500
Erk1/2	Cell Signaling, #9107	1:1000
Phosphorylated Erk1/2 (Thr202/Tyr204)	Cell Signaling, #4379	1:1000
AKT	Cell Signaling, #4691	1:1000
Phosphorylated AKT (Ser473)	Cell Signaling, #4060	1:1000
Actin, mouse monoclonal	Abcam, MAB1501	1:1000
Peroxidase-labeled anti-mouse	Promega, W402B	1:10,000
Peroxidase-labeled anti-rabbit	Promega, W401B	1:10,000

### Analysis of the bone marrow vasculature

To determine whether the bone marrow vasculature is altered in a Tgif1-deficient bone microenvironment, the number (/mm^2^ tissue area), length (mm), and size (mm^2^) of the Endomucin-positive bone marrow vasculature were analyzed on 3–4 non-serial, gelatin-embedded sections of the tibiae from mice that were sacrificed 5 days after tumor cell injection. Only mice without tumor cells were used for quantification. The analysis was performed as outlined in Additional file 1: Figure S1A using the Osteomeasure software and an Olympus BX50 microscope (Olympus UPlan Fl 10x/0.30 ∞/-). Briefly, an area of 1125 mm^2^ in the metaphysis was quantified starting 180 μm away from the growth plate and 225 μm away from the medial cortex.

### Micro-computed tomography

For micro-computed tomography (μCT), the tibiae were scanned during tissue fixation either in 4% PFA or in 70% ethanol at later time points. Trabecular bone volume (bone volume per total volume, BV/TV), trabecular number (Tb.N), trabecular thickness (Tb.Th), and trabecular separation (Tb.Sp) were analyzed using a vivaCT 80 scanner (Scanco Medical). Bones were scanned at 70 kVp with a pixel size of 15.6 μm. All trabecular bone surfaces 78 μm away from the growth plate were analyzed with a length of 1 mm of the region of interest. Following the acquisition of the grayscale images, images were converted into binary images with thresholds being consistent within one study, and bone parameters were calculated using the proprietary scanner software.

### Immunoblot analysis

Whole-cell protein lysates were obtained using lysis buffer (150 nM NaCl, 0.5% NP-40, 0.25% sodium deoxycholate, 50 mM Tris pH 7.5) supplemented with a protease inhibitor (Roche Diagnostics, 11873580001) and a phosphatase inhibitor (Roche Diagnostics, 4906837001). Protein concentration was quantified using the Pierce™ BCA Protein Assay Kit (Thermo Fisher, 23225) according to the manufacturer’s protocol. Lysates were separated on a 12% acrylamide gel and immobilized onto nitrocellulose blotting membranes (Amersham Protran®, 0.2 μm NC, Roth) using a Tans-Blot Turbo Transfer System (30 min, 25 V, BioRad). The membranes were blocked using 5% skimmed milk (Roth, Art Nr.T145.2) for 1 h at room temperature before overnight incubation at 4 °C with primary antibodies against Tgif1 (1:500, rabbit monoclonal antibody, Abcam, ab52955), Erk1/2 (1:1000, mouse monoclonal antibody, Cell Signaling, #9107), phosphorylated Erk1/2 (Thr202/Tyr204) (1:1000, rabbit monoclonal antibody, Cell Signaling, #4379), AKT (1:1000, rabbit polyclonal antibody, Cell Signaling, #4691), and phosphorylated AKT (Ser473) (1:1000, rabbit monoclonal antibody, Cell Signaling, #4060). Immunoblot for actin was used as a loading control (1:1000, mouse monoclonal antibody, Abcam, MAB1501). Secondary antibodies were incubated for 1 h at room temperature, followed by signal detection using the Clarity Western ECL Substrate (BioRad) and the ChemiDoc™ MP Imaging System (Bio-Rad).

### RNA extraction and gene expression analysis

Total RNA was isolated from cultured cells using the RNeasy Plus Mini Kit (Qiagen, 74136) according to the manufacturer’s protocol. After sacrifice, the lungs were snap-frozen in liquid nitrogen and stored at − 80 °C. Total RNA was isolated from the lungs using TRIzol reagent (Sigma, T9424) according to the manufacturer’s instructions. cDNA was synthesized using 1 μg RNA and the ProtoScript First Strand cDNA Synthesis Kit (E6300S). Quantitative real-time PCR (qRT-PCR) was performed with the CFX Connect Real-Time PCR Detection System (Bio-Rad) using the SYBR™ Select Master Mix for CFX (Applied Biosystems, 4472942). Data were normalized to the expression of the housekeeping genes glycerinaldehyde-3-phosphate-dehydrogenase (GAPDH) or beta-2-microglobulin (B2M, Table 2). GFP expression was used to determine the presence of tumor cells in the lungs 5 days after tumor cell injection.

**Table 2:** Oligonucleotide sequences used in this study

Target gene	Sense	Antisense
mGFP	CAGGAGCGCACCATCTTCTT	CTCGATGTTGTGGCGGATCT
mB2M	CTGCTACGTAACACAGTTCCACCC	CATGATGCTTGATCACATGTCTCG
mTgif1	GAGGATGAAGACAGCATGGA	TTCTCAGCATGTCAGGAAGG
mSema3E	GGGGCAGATGTCCTTTTGA	AGTCCAGCAAACAGCTCATTC
mGAPDH	TCACCACCATGGAGAAGGC	GCTAAGCAGTTGGTGGTGCA

### Histological analysis

For the analysis of bone volume, MMA-embedded sections were stained with von Kossa/van Gieson staining. To identify osteoblasts and osteoclasts, bone sections were stained using toluidine blue and tartrate-resistant acid phosphatase (TRAP) staining, respectively. Bone cell number/mm trabecular bone surface as well as dynamic histomorphometric parameters were analyzed using the OsteoMeasure software attached to an Olympus BX50 microscope, × 20 objective (Olympus UPlan Fl 20x/0.50 ∞/0.17) according to the standards of the American Society for Bone and Mineral Research (ASBMR) [37]. The tumor volume and the trabecular bone volume in the femora of mice were quantified using hematoxylin and eosin- or van Giemsa-stained sections (2 non-serial sections per mouse, 20 μm apart) 9 days after tumor cell injection. The analysis was performed using the OsteoMeasure software and an Olympus BX50 microscope (× 10 objective, Olympus UPlan Fl 10x/0.30 ∞/-). The total tumor area and the trabecular bone area (Additional file 1: Figure S1B) of a tissue area with a length of 2700 μm were quantified.

### Enzyme-linked immunosorbent assay

Blood was collected by cardiac puncture and allowed to coagulate at room temperature, followed by centrifugation at 6200*g* for 10 min. The serum was collected and stored at − 80 °C until quantification of the bone formation marker pro-collagen type I N propeptide (P1NP, Immunodiagnostic Systems, AC-33F1) and of the bone resorption marker tartrate-resistant acid phosphatase (TRAP, Immunodiagnostic Systems, SB-TR103). ELISA analyses were performed according to the manufacturer’s instructions.

### Statistical analyses

Statistical analyses were performed using the Prism GraphPad software (Version 8.0.1). Data were analyzed using Student’s *t* test when comparing two groups or by one-way analysis of variance (ANOVA), followed by Tukey’s post hoc test when comparing more than two groups. The applied test is indicated in each figure legend with a *p* value < 0.05 being considered as statistically significant.

## Results

### Tgif1 supports the osteoblast-mediated increase of breast cancer cell migration

**Fig. 1:**
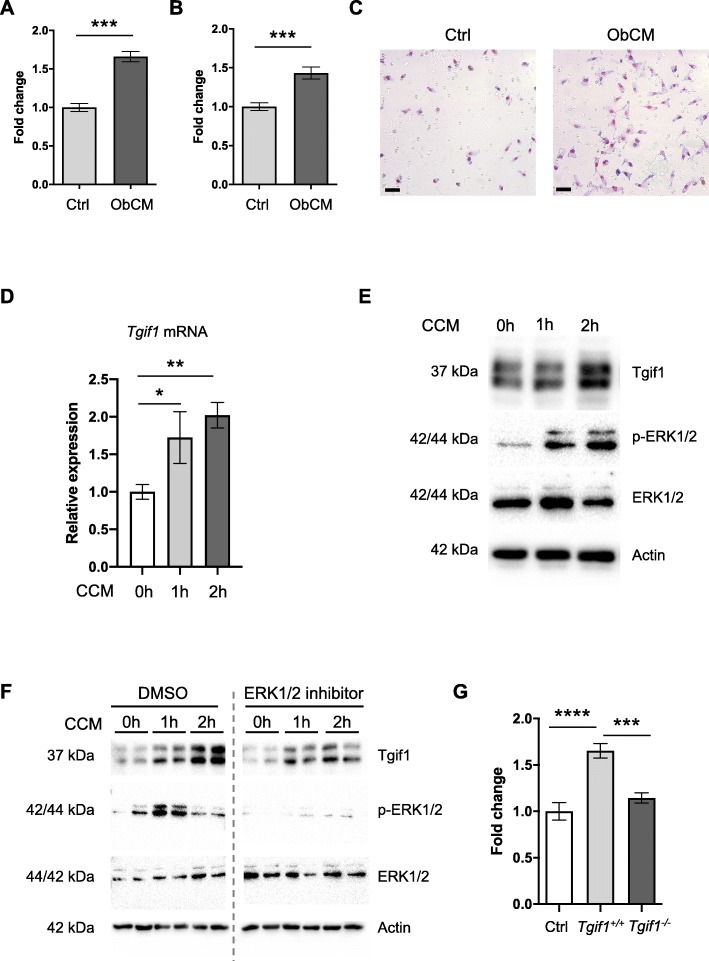
Tgif1 supports the osteoblast-breast cancer cell interaction in vitro*.***a** Migration of 4T1-GFP and **b** of MDA-MB-231 breast cancer cells towards control (Ctrl) medium or medium conditioned by osteoblasts (ObCM). **c** Representative images of MDA-MB-231 cells immobilized on transwell inserts after migration. Scale bar indicates 20 μm. **d** Tgif1 mRNA expression in osteoblasts 1 and 2 h after stimulation with medium conditioned by 4T1 breast cancer cells (CCM). **e** Immunoblot demonstrating the protein abundance of Tgif1, phosphorylated ERK1/2 (p-ERK1/2), and total ERK1/2 in MC3T3-E1 osteoblasts after stimulation with CCM for 1 and 2 h. Immunoblot for actin was used as a loading control. **f** MC3T3-E1 cells were incubated with vehicle (DMSO) or with an inhibitor of ERK1/2 signaling and stimulated with CCM for 1 and 2 h. Immunoblot to demonstrate the abundance of Tgif1, p-ERK1/2, total ERK1/2, and actin. **g** Transwell assay of MDA-MB-231 breast cancer cell migration towards Ctrl medium or medium conditioned by osteoblasts that were isolated from mice bearing a germ line deletion of Tgif1 (*Tgif1*^*−/−*^) or from *Tgif1*^*+/+*^ control littermates. *N* = 3–5 with experimental replicates. Data are presented as mean ± SEM. Two-tailed Student’s *t* test was used to compare two groups (**a**, **b**), and ANOVA followed by Tukey’s post hoc analysis was used to compare more than two groups (**d**, **g**); *p < 0.05, **p < 0.001, ***p < 0.001, ****p < 0.0001

Patients with breast cancer bone metastases often present with osteolytic lesions, and therefore, osteoclasts are considered as the cellular drivers of the disease. However, osteoblasts have recently been proposed as potential early-stage mediators of bone metastasis progression [19, 38]. Yet, very limited knowledge exists about the role of osteoblasts in initiating destructive bone lesions. We hypothesized that osteoblasts regulate early stages of breast cancer bone metastases, including the migration of breast cancer cells to the metastatic site. To test this hypothesis in vitro, we used transwell migration assays allowing breast cancer cells to migrate towards the medium that had been conditioned by osteoblasts. Indeed, osteoblast-conditioned medium stimulated the migration of both cells of the mouse-derived 4T1 (Fig. 1a) and of the human-derived MDA-MB-231 (Fig. 1b, c) breast cancer cell lines, suggesting that osteoblasts attract breast cancer cells to the metastatic site.

Next, we aimed to identify the molecular mediators of breast cancer cell-osteoblast interaction. Recently, we identified Tgif1 as a novel target gene of the first 34 amino acids of human recombinant parathyroid hormone (PTH 1-34) [32]. PTHrP is a key cancer cell-derived cytokine and activates, like PTH 1-34, the PTH1R receptor [39–41]. We therefore hypothesized that PTHrP might also increase Tgif1 expression in osteoblasts and stimulated MC3T3-E1 cells with PTHrP. Indeed, PTHrP treatment significantly increased Tgif1 mRNA expression in osteoblasts (Additional file 2: Figure S2A). Consistently, immunoblot analysis revealed a strong increase in Tgif1 expression 6 h after stimulation with PHTrP (Additional file 2: Figure S2B). This finding lets us to propose that CCM might also stimulate Tgif1 expression in osteoblasts. In support of this hypothesis, CCM increased Tgif1 mRNA expression already 1 h after stimulation (Fig. 1d), suggesting that CCM stimulates Tgif1 expression at the transcriptional level. The expression continued to increase after 2 and 6 h of CCM stimulation (Fig. 1d, Additional file 2: Figure S2C). Consistently, immunoblot analysis demonstrated an increase in Tgif1 expression in osteoblasts as early as 1 h after stimulation with CCM, with a sustained increase of Tgif1 expression after 2 and 6 h of exposure to CCM (Fig. 1e, Additional file 2: Figure S2D).

To investigate signaling cascades that might be involved in the CCM-induced increase of Tgif1 expression in osteoblasts, we determined the activation of various signaling pathways involved in cell migration including AKT and ERK1/2 signaling. Interestingly, CCM stimulation activated the ERK1/2 pathway while no activation of the AKT pathway was observed (Fig. 1e, Additional file 2: Figure S2E). To test the hypothesis that the increase in Tgif1 expression depends on the activity of the ERK1/2 signaling cascade, osteoblasts were treated with a selective ERK1/2 inhibitor prior to the stimulation with CCM. Inhibition of the ERK1/2 pathway impaired the CCM-induced increase of Tgif1 expression, suggesting that CCM stimulates Tgif1 expression in part through the ERK1/2 pathway (Fig. 1f).

To investigate whether Tgif1 in osteoblasts is involved in regulating the osteoblast-mediated increase in breast cancer cell migration, we induced breast cancer cell migration with conditioned medium collected from osteoblasts that were isolated from mice bearing a germ line deletion of Tgif1 (*Tgif1*^*−/−*^) or from control littermates (*Tgif1*^*+/+*^). Consistent with our findings using the MC3T3-E1 cell line, medium conditioned by *Tgif1*^*+/+*^ primary osteoblasts significantly increased breast cancer cell migration compared to control (Fig. 1g). In contrast, medium conditioned by *Tgif1*^*−/−*^ osteoblasts failed to increase the migration of MDA-MB-231 breast cancer cells (Fig. 1g). These findings strongly indicate that Tgif1 is required for the osteoblast-mediated increase of breast cancer cell motility.

### Tgif1 deficiency reduces the formation of bone marrow micro-metastases

**Fig. 2:**
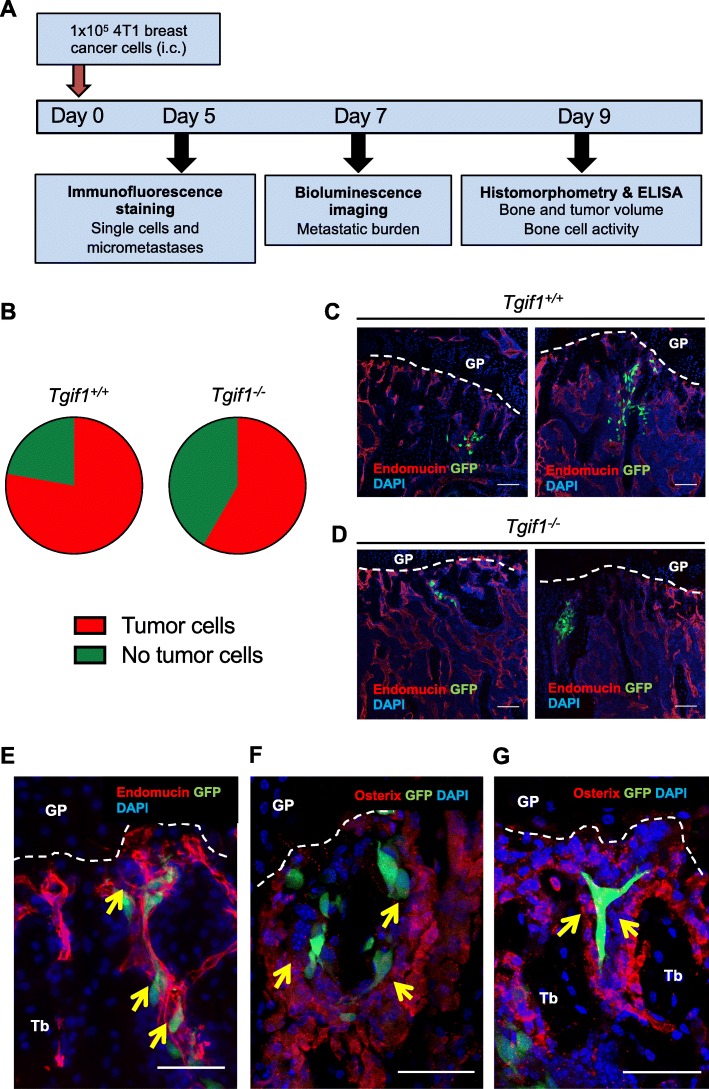
Tgif1 deficiency reduces the presence of single tumor cells and micro-metastases in the bone microenvironment. **a** Schematic presentation of the experimental outline to determine the role of Tgif1 during the establishment and progression of breast cancer bone metastases in 8–10-week-old female BALB/c mice with a germ line deletion of Tgif1 (*Tgif1*^*−/−*^) or control littermates (*Tgif1*^*+/+*^). i.c., intra cardiac injection. **b** Presence of single tumor cells and micro-metastases in the tibiae 5 days after tumor cell injection (*n* = 9 for *Tgif1*^*+/+*^, *n* = 12 for *Tgif1*^*−/−*^). **c**, **d** Representative confocal images of gelatin-embedded tibiae from *Tgif1*^*+/+*^ (**c**) and *Tgif1*^*−/−*^ (**d**) mice stained for the vascular endothelial cell marker Endomucin (red) and breast cancer cells (green). GP, growth plate. Scale bar indicates 100 μm. **e**–**g** Representative images showing the proximity of breast cancer cells (green) to Endomucin-positive vascular endothelial cells (red) (**e**) and Osterix-positive osteoblasts (red) (**f**, **g**) is indicated by yellow arrowheads. Tb, trabecular bone. Scale bar indicates 50 μm

Our in vitro findings suggest that Tgif1 is important for the increase of breast cancer cell migration upon stimulation with the medium that had been conditioned by osteoblasts, raising the question whether Tgif1 might also be implicated in the initiation of metastatic bone disease in vivo*.* To test this hypothesis, we employed a syngeneic metastasis model using *Tgif1*^*−/−*^ and *Tgif1*^*+/+*^ mice (Fig. 2a). In support of our hypothesis, immunofluorescence staining and confocal microscopy revealed that 5 days after breast cancer cell injection, the presence of tumor cells in the bone marrow microenvironment was reduced by 25% in *Tgif1*^*−/−*^ mice compared to *Tgif1*^*+/+*^ mice (Fig. 2b). While breast cancer cells were found in approximately 78% of *Tgif1*^*+/+*^ mice, only 58% of *Tgif1*^*−/−*^ mice had breast cancer cells in the bone marrow. At this time point, we observed single tumor cells and breast cancer micro-metastases in the tibiae of both groups, albeit less frequent in the absence of Tgif1 (Fig. 2c, d). Immunofluorescence staining of the bone marrow vasculature using the endothelial cell marker Endomucin and the osteoblast marker Osterix revealed that tumor cells preferentially localize in close proximity to both vessels and osteoblasts (Fig. 2e–g). To determine if the deletion of Tgif1 affects tumor cell homing to organs other than bones, we performed a qRT-PCR analysis to detect GFP-positive breast cancer cells in the lungs of *Tgif1*^*+/+*^ and *Tgif1*^*−/−*^ mice. A GFP signal was only detected in the lungs of a small subset of mice and did not differ between genotypes (Additional file 2: Figure S2A and S2B). This suggests that the reduced number of micro-metastases in the bone marrow microenvironment is not due to tumor cell relocation to other organs. Together, these findings demonstrate that Tgif1 plays an important positive role during the early stages of breast cancer bone metastasis, which might be regulated by osteoblasts and vascular endothelial cells.

### Metastatic breast cancer burden is reduced in a Tgif1-deficient bone microenvironment

**Fig. 3:**
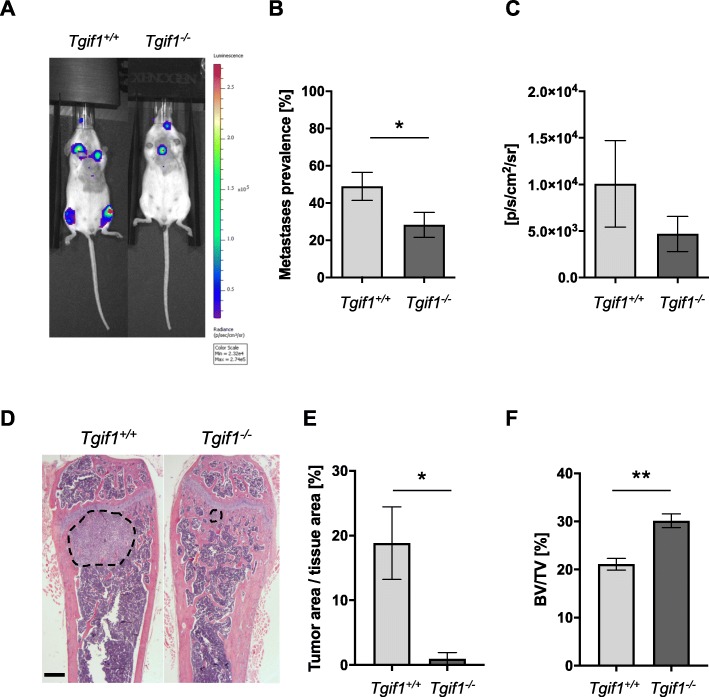
Metastatic breast cancer burden is reduced in a Tgif1-deficient bone microenvironment in vivo*.***a**, **b** Bioluminescence imaging and prevalence of bone metastases 7 days after intracardiac injection (*n* = 44–46 long bones/group). **c** Bioluminescence signal quantification shown as average radiance (p/s/cm^2^/sr) in *Tgif1*^*+/+*^ and *Tgif1*^*−/−*^ mice (*n* ≥ 20 mice/group). **d**–**f** Representative images of hematoxylin and eosin-stained femurs (**d**) and quantification of tumor volume (tumor area/tissue area) (**e**) and trabecular bone volume (BV/TV; bone volume/tissue volume) (**f**) 9 days after tumor cell injection (*n* = 4–7 mice/group). Scale bar indicates 1 mm. Data are presented as mean ± SEM. Two groups were compared using two-tailed Student’s *t* test (**b**, **c**, **f**) or Welch’s *t* test (**e**); *p < 0.05, **p < 0.01

To determine whether the reduced presence of single cells and breast cancer micro-metastases 5 days after tumor cell injection results in a decreased metastatic burden, we analyzed the establishment of bone metastases in *Tgif1*^*−/−*^ and *Tgif1*^*+/+*^ mice. Supporting our hypothesis, bioluminescence imaging revealed a decreased number of bone metastases in the long bones of *Tgif1*^*−/−*^ mice compared to control littermates (28.26% vs. 48.89%, respectively, Fig. 3a, b) 7 days after tumor cell injection. In addition, the reduced bioluminescence signal intensity is consistent with an attenuated metastatic growth in the long bones of *Tgif1*^*−/−*^ mice (Fig. 3a, c). However, this did not reach statistical significance due to a high variance of the data. Nevertheless, in support of our hypothesis, histological analysis revealed a decreased tumor volume per tissue volume in the femora of *Tgif1*^*−/−*^ mice 9 days after tumor cell injection (Fig. 3d, e). Consistently, trabecular bone volume was significantly higher in the femora of *Tgif1*^*−/−*^ mice compared to *Tgif1*^*+/+*^ mice (Fig. 3f), suggesting that the lack of Tgif1 protects from breast cancer-induced bone loss. This is supported by a reduced concentration of TRAP5b and an increased concentration of PINP in the serum obtained from *Tgif1*^*−/−*^ mice (Additional file 3: Figure S3C and S3D). In summary, these data suggest that the absence of Tgif1 reduces the bone metastatic burden and protects from breast cancer-induced bone destruction in vivo*.*

### Tgif1 maintains the osteoblast activity in the bone marrow microenvironment

**Fig. 4:**
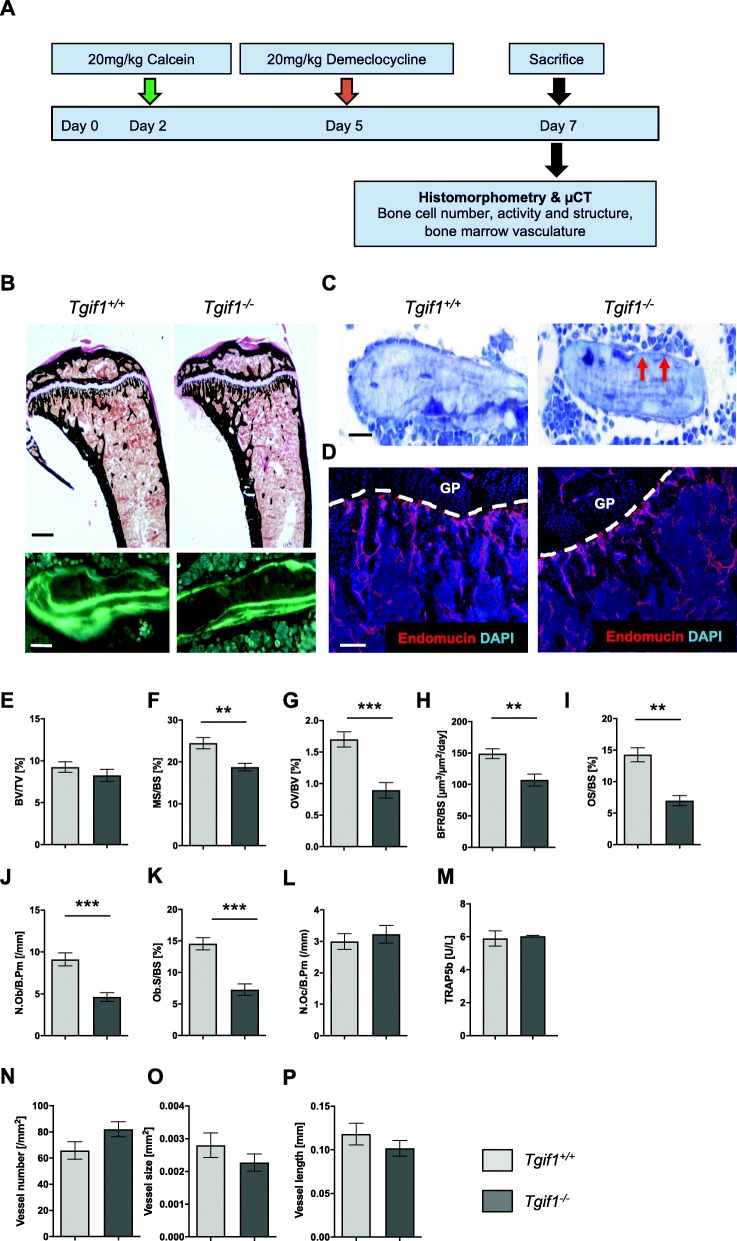
Absence of Tgif1 in the bone microenvironment reduces the number and activity of osteoblasts in vivo*.***a** Schematic presentation of the experimental outline to investigate the role of Tgif1 in the bone microenvironment using 8-week-old mice with a germ line deletion of Tgif1 (*Tgif1*^*−/−*^) or control littermates (*Tgif1*^*+/+*^). **b** Representative images of von Kossa-stained sections of the tibiae (top panel) and of fluorescence double labeling (lower panel) from *Tgif1*^*+/+*^ and *Tgif1*^*−/−*^ mice. Scale bars indicate 1 mm (black) and 20 μm (white). **c** Representative images of toluidine blue-stained sections of the tibiae demonstrating the reduction of osteoblasts (red arrows) lining trabecular bone surfaces in *Tgif1*^*−/−*^ mice compared to *Tgif1*^*+/+*^ mice. Scale bar indicates 20 μm. **d** Representative images of the bone marrow vasculature. Tibia sections from *Tgif1*^*+/+*^ and *Tgif1*^*−/−*^ mice were stained for Endomucin to visualize vessels. GP, growth plate. Scale bar indicates 100 μm. **e**–**l** Histomorphometric analysis of trabecular bone mass (**e**), mineralized bone surfaces (**f**), osteoid volume (**g**), bone formation rate (**h**), osteoid surface (**i**), osteoblast number (**j**), osteoblast surface (**k**), and the number of osteoclasts (**l**) in *Tgif1*^*+/+*^ and *Tgif1*^*−/−*^ mice (*n* = 5–10 mice/group). BV/TV, bone volume/tissue volume; MS/BS, mineralizing surface/bone surface; OV/BV, osteoid volume/bone volume; BFR/BS, bone formation rate/bone surface; OS/BS, osteoid surface/bone surface; N.Ob/BS, number of osteoblasts/bone surface; Ob.S/BS, osteoblast surface/bone surface; N.Oc/BS, number of osteoclasts/bone surface. **m** Serum TRAP5b concentration measured by enzyme-linked immunosorbent assay (ELISA) (*n* = 3–6 mice/group). **n**–**p** Number (**n**), size (**o**), and length (**p**) of Endomucin-positive bone marrow vessels were analyzed using 3–4 non-serial sections of the tibiae per mouse. Data are presented as mean ± SEM. Two-tailed Student’s *t* test was used to compare the two groups; **p < 0.01, ***p < 0.001

In order to investigate which components of the bone microenvironment might contribute to the reduced metastatic burden in Tgif1-deficient mice, we performed a detailed characterization of the bone microenvironment of 8-week-old female *Tgif1*^*−/−*^ mice and control littermates (Fig. 4a). μCT analysis revealed that deletion of Tgif1 did not affect the trabecular bone mass (bone volume/total volume (BV/TV)), trabecular thickness (Tb.Th), trabecular number (Tb.N), or the trabecular separation (Tb.Sp) in the tibiae of tumor-free mice (Additional file 4: Figure S4A-D) or of mice that were sacrificed 5 days after tumor cell injection (Additional file 4: Figure S4E-H). These data were confirmed by histomorphometric analysis, demonstrating no change of the trabecular bone mass (Fig. 4b (upper panel), e). Since tumor cells were found to localize in close proximity to Osterix-positive osteoblasts lining the trabecular bone surfaces at early stages of metastasis formation (Fig. 2f, g), we investigated the number and function of osteoblasts in greater detail. Dedicated histomorphometric analysis revealed a significant reduction of the mineralizing surface per bone surface (MS/BS), the osteoid volume per bone volume (OV/BV), the bone formation rate per bone surface (BFR/BS), and the osteoid surface per bone surface (OS/BS) in *Tgif1*^*−/−*^ mice compared to control littermates (Fig. 4b (lower panel), c, f–i). These data indicate a reduced osteoblast activity in the absence of Tgif1. Indeed, the number of osteoblasts per trabecular bone surface (N.Ob/B.Pm) and the osteoblast surface per bone surface were significantly reduced in *Tgif1*^*−/−*^ mice compared to control littermates (Fig. 4c, j, k). In contrast, the number of osteoclasts per bone surface (N.Oc/B.Pm) (Fig. 4l) and the osteoclast activity, determined by the quantification of the TRAP5b serum concentration (Fig. 4m), were not altered by the absence of Tgif1.

Since single breast cancer cells localize not only close to osteoblasts but also nearby Endomucin-positive vascular endothelial cells (Fig. 2e), we investigated potential alterations of the bone marrow vasculature in non-tumor cell-bearing tibiae from *Tgif1*^*+/+*^ and *Tgif1*^*−/−*^ mice. No difference in the number, size, and length of the bone marrow vasculature was found between *Tgif1*^*+/+*^ and *Tgif1*^*−/−*^ mice (Fig. 4d, n–p). These findings suggest that the reduced incidence of bone metastases in *Tgif1*^*−/−*^ mice might be independent of the bone marrow vasculature and, at least in part, be mediated by active osteoblasts.

### Elevated expression of Semaphorin 3E in the absence of Tgif1 impairs breast cancer cell migration

**Fig. 5:**
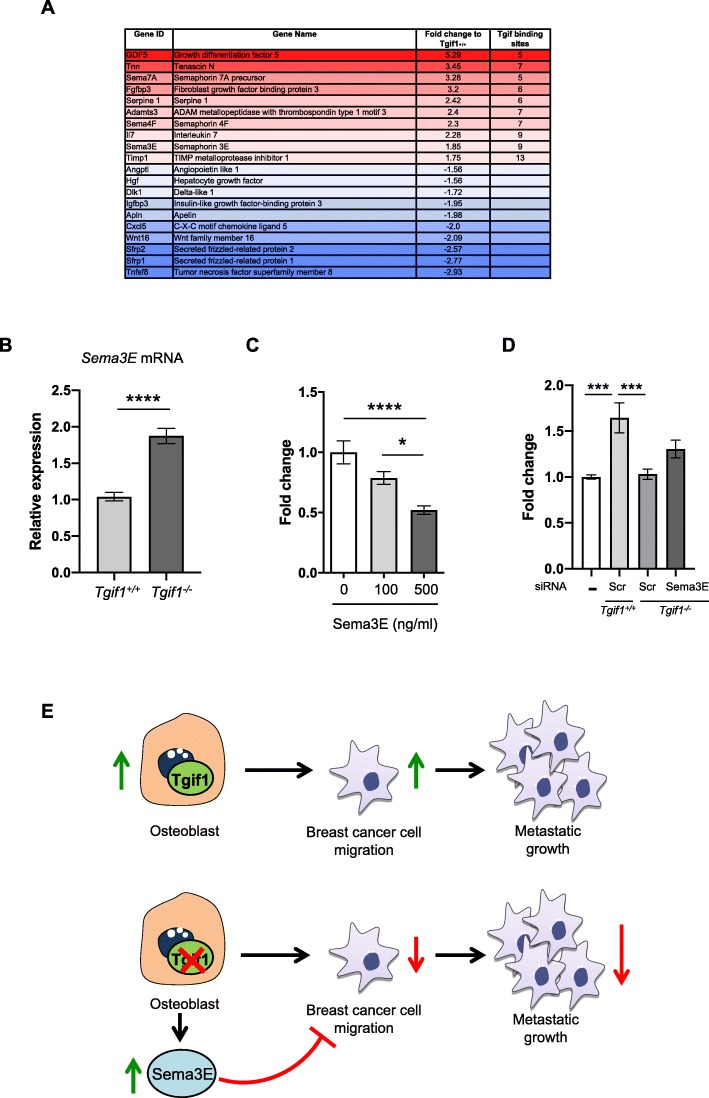
Tgif1 supports breast cancer cell migration by suppressing Sema3E expression in osteoblasts. **a** RNA sequencing-based fold expression of target genes in *Tgif1*^*−/−*^ osteoblasts compared to osteoblasts from *Tgif1*^*+/+*^ control littermates. The number of predicted Tgif binding sites on the 3-kb proximal promoter of upregulated transcripts is indicated in the right column. **b** Semaphorin 3E (Sema3E) mRNA expression in *Tgif1*^*−/−*^ and *Tgif1*^*+/+*^ osteoblasts. **c** MDA-MB-231 breast cancer migration towards increasing concentrations of recombinant Sema3E (0–500 ng/ml). **d** Migration of 4T1 breast cancer cells towards control medium and medium conditioned by *Tgif1*^*+/+*^ osteoblasts transfected with scrambled control siRNA (Scr) and *Tgif1*^*−/−*^ osteoblasts transfected with Scr or siRNA against Sema3E (siSema3E), *n* = 6 independent experiments. **e** Schematic presentation of the positive role of Tgif1 in osteoblasts in supporting breast cancer cell migration and metastatic growth. Data are presented as mean ± SEM. Two-tailed Student’s *t* test was used to compare two groups (**b**), and ANOVA followed by Tukey’s post hoc analysis was used to compare more than two groups (**c**, **d**); *p < 0.05, ***p < 0.001, ****p < 0.0001

To better understand the molecular mechanism underlying the reduced metastatic burden in *Tgif1*^*−/−*^ mice, we performed an unbiased RNA-seq analysis using osteoblasts obtained from *Tgif1*^*+/+*^ and *Tgif1*^*−/−*^ mice (Fig. 5a). Subsequently, we performed an in silico analysis for predicted Tgif binding sites in the proximal promoters of the ten most abundantly expressed mRNAs that encode secreted proteins using the TRANSFAC database (Fig. 5a). Furthermore, we searched for factors that were regulated at the mRNA and protein level using a published secretome dataset (Stable Isotope Labeling with Amino Acids in Cell Culture (SILAC), ProteomeXchange dataset, identifier PXD012303). From these investigations, we selected Semaphorin 3E (Sema3E) for further analysis since it is abundantly expressed by *Tgif1*^*−/−*^ osteoblasts, has nine Tgif binding sites in the proximal promoter, and is secreted by osteoblasts. The difference in Sema3E expression between *Tgif1*^*+/+*^ and *Tgif1*^*−/−*^ osteoblasts was subsequently confirmed by RT-qPCR analysis (Fig. 5b). Since Sema3E has been shown to inhibit the migration of various cell types [42–44], we hypothesized that Sema3E could also restrict the motility of breast cancer cells. To test this hypothesis, we allowed breast cancer cells to migrate towards increasing concentrations of Sema3E (100 ng/ml and 500 ng/ml). In support of our concept, recombinant Sema3E dose-dependently impaired breast cancer cell migration (Fig. 5c). Importantly, conditioned medium collected from *Tgif1*^*−/−*^ osteoblasts transfected with siRNA against Sema3E partially but significantly restored breast cancer cell migration to the level of medium conditioned by *Tgif1*^*+/+*^ osteoblasts (Fig. 5d). Together, these results suggest that Tgif1 in osteoblasts supports breast cancer cell migration by suppressing Sema3E expression.

## Discussion

In this study, we identified Tgif1 as a novel regulator of the osteoblast-breast cancer cell interaction. We propose that the lack of Tgif1 in osteoblasts attenuates breast cancer cell migration and metastasis formation, presumably through suppression of Sema3E expression (Fig. 5e). Thus, our findings establish osteoblasts and Tgif1 as important regulators of bone metastasis.

The metastatic cascade is a multistep process, and in particular, migration and invasion are considered as hallmarks of cancer malignancy [45]. For successful colonization of distant organs, an interaction between tumor cells and the local environment is important. Here, we demonstrate that osteoblasts mediate the chemotactic migration of breast cancer cells in vitro. In support of our results, medium conditioned by pre-osteoblasts was recently demonstrated to stimulate collective cell migration of metastatic breast cancer cells in a wound healing assay [46]. Together, these findings suggest that osteoblasts attract breast cancer cells to the metastatic site, thereby playing a key role during the formation of bone metastases.

Tgif1 has been identified as a stimulator of cancer cell migration as shown by an enhanced migration of colon cancer cells upon overexpression of Tgif1 [30]. Consistently, the absence of Tgif1 impaired the migration of non-small lung cell cancer cells and MDA-MB-231 breast cancer cells in vitro [47, 48]. In breast cancer, long-term exposure to the carcinogen cadmium was shown to promote breast cancer cell migration and invasion by increasing the expression of Tgif1 [49]. Thus, these studies indicate a cell-autonomous effect of Tgif1 in stimulating breast cancer cell migration. Here, we aimed to investigate whether Tgif1 in osteoblasts could participate in regulating the osteoblast-induced breast cancer cell migration. Indeed, medium conditioned by control osteoblasts significantly stimulated the migration of breast cancer cells, while medium conditioned by osteoblasts lacking Tgif1 failed to activate breast cancer cell migration. Hence, our data show for the first time that Tgif1 in osteoblasts supports breast cancer cell migration in a non-cell-autonomous manner.

To obtain further insights into the molecular mechanisms underlying the role of Tgif1 in the osteoblast-mediated breast cancer cell migration, we performed an unbiased RNA sequencing analysis in osteoblasts isolated from *Tgif1*^*+/+*^ and *Tgif1*^*−/−*^ mice. In this screen, Sema3E, a class 3 Semaphorin, was identified as abundantly expressed by *Tgif1*^*−/−*^ osteoblasts. Class 3 Semaphorins comprise a group of secreted molecules that were originally described as chemorepulsive molecules regulating axon guidance [50, 51]. Besides their function in the nervous system, class 3 Semaphorins have been shown to restrict cell migration in a variety of biological systems in a context-dependent manner [52]. For instance, Sema3E plays an important role in leukocyte trafficking by inhibiting inflammation-induced neutrophil migration and recruitment to the lungs [53]. Furthermore, migration of vascular smooth muscle cells, osteoblasts, and thymocytes is inhibited by Sema3E [54–56]. In addition, Sema3E produced by immature dendritic cells inhibits the migration of natural killer cells, demonstrating the role of Sema3E in regulating cell-cell interaction [57]. Consistently, our findings suggest that osteoblast-derived Sema3E suppresses breast cancer cell migration, providing a novel paracrine function for Sema3E.

Besides stimulating breast cancer cell migration, an increased abundance of Tgif1 has been associated with mammary tumorigenesis [31]. In support of this finding, knockdown of Tgif1 in MDA-MB-231 breast cancer cells reduced the presence of lung metastases in mice [48], suggesting that Tgif1 promotes cell-autonomous breast cancer growth and metastasis. While these studies established the role of Tgif1 in breast cancer cells, we determined whether Tgif1 in the tumor microenvironment affects the progression of bone metastases in a non-cell-autonomous manner. Interestingly, the deletion of Tgif1 in the bone microenvironment reduced the presence of single breast cancer cells and breast cancer micro-metastases in the bone. Furthermore, metastatic growth was attenuated in a Tgif1-deficient bone microenvironment, resulting in a reduced breast cancer-mediated bone destruction. Recently, it has been proposed that static environments (i.e., endosteal surfaces covered by lining cells, stable vasculature) maintain disseminated tumor cells quiescent, while active environments (i.e., sprouting vasculature) trigger tumor cell growth in bones [20]. Thus, modifying the bone microenvironment might offer promising therapeutic approaches to restrict tumor growth in bone [58].

As components of the heterogeneous bone microenvironment, the vascular and endosteal niches are of particular importance for metastatic breast cancer growth in bones [16, 38, 59]. Our dedicated analysis of the vascular network revealed no alterations of the number, length, or size of the Endomucin-positive vasculature in the long bones of Tgif1-deficient mice. Previously, it has been shown that silencing Tgif1 expression decreased the proliferation while it increased the tube formation of endothelial cells in vitro [60]. In addition, an increased angiogenic potential as determined by vascular network formation assays was observed upon silencing of Tgif1 expression [60]. However, our results indicate that these in vitro findings do not translate into in vivo conditions and cause changes in the mineralized surface, bone marrow vasculature. In contrast, the number and activity of osteoblasts including the bone formation rate, osteoid volume, and surface were significantly reduced in *Tgif1*^*−/−*^ mice compared to control littermates. This suggests that the attenuated metastatic burden in Tgif1-deficient mice is, at least in part, mediated by osteoblasts rather than by the bone marrow vasculature. In vitro, Tgif1-deficient osteoblasts reduced breast cancer cell migration in a Sema3E-dependent manner. It is therefore likely that Tgif1 in osteoblasts also regulates breast cancer cell migration to the metastatic site in vivo. However, the strong reduction of the number of active osteoblasts suggests that additional mechanisms independent of cell migration may exist that control osteoblast function and consequently breast cancer cell proliferation and disease progression in vivo. While the experimental approaches used in this study do not allow distinguishing between these possibilities, the contribution of the migration-stimulating effect of Tgif1 and the effect on osteoblast activity would need to be elucidated in the future. Furthermore, to better understand which step of the metastatic cascade Tgif1 controls precisely, additional in vivo models could be employed. In the present study, a syngeneic mouse model was chosen to preserve an intact immune system, which has an important role in bone metastasis progression. However, the 4T1 breast cancer cells grow very aggressively in the bone, which makes it challenging to distinguish between the different steps of disease progression such as cancer cell homing, dormancy, micrometastasis formation, and relapse. Therefore, future experiments may include less aggressive xenograft models that recapitulate the corresponding clinical situation more closely.

In summary, this work demonstrates that Tgif1 in the bone microenvironment is implicated in the establishment and progression of breast cancer bone metastases and might therefore provide novel therapeutic opportunities to treat the initiation and progression of breast cancer metastasis to bones.

## Conclusions

Breast cancer bone metastases are characterized by an increased osteoclast-mediated bone resorption. Due to the osteolytic nature of the disease, osteoclasts have been considered as the cellular drivers of bone metastases while little is known about bone-forming osteoblasts in this process. Here, we show that osteoblasts and breast cancer cells functionally interact in vitro and in vivo. We identified the homeodomain protein Tgif1 as an important mediator of this interaction and demonstrate that bone metastases burden is reduced in the absence of Tgif1 in the bone marrow microenvironment. In conclusion, our results suggest that Tgif1 in osteoblasts is an important regulator of breast cancer cell motility, and its presence in the bone microenvironment affects metastasis formation in the skeleton.

## Supplementary information

**Additional file 1: Supplemental Figure 1.** Illustration of the histological analysis.

**Additional file 2: Supplemental Figure 2.** PTHrP and cancer conditioned medium increase Tgif1 expression in osteoblasts in vitro*.*

**Additional file 3: Supplemental Figure 3.** Analysis of tumor cell distribution and bone turnover markers in cancer-bearing mice.

**Additional file 4: Supplemental Figure 4.** Analysis of the bone phenotype of *Tgif1+/+* and *Tgif1-/-* mice.

## Data Availability

All data generated or analyzed during this study are included in this published article and its supplementary information files.

## Abbreviations

RANKL: Receptor activator of NF-κB ligand
Tgif1: TG-interacting factor-1
PTHrP: Parathyroid hormone-related protein
CM: Conditioned medium
CCM: Cancer-conditioned medium
Sema3E: Semaphorin 3E
ObCM: Osteoblast-conditioned medium
Ctrl: Control
GFP: Green fluorescence protein
TRAP: Tartrate-resistant acid phosphatase
PINP: Procollagen type I N propeptide

## References

[1] Lundqvist A, Andersson E, Ahlberg I, Nilbert M, Gerdtham U (2016). Socioeconomic inequalities in breast cancer incidence and mortality in Europe-a systematic review and meta-analysis. Eur J Pub Health.

[2] Society AC (2019). Cancer facts & figures 2019.

[3] Coleman RE, Smith P, Rubens RD (1998). Clinical course and prognostic factors following bone recurrence from breast cancer. Br J Cancer.

[4] Coleman RE (2001). Metastatic bone disease: clinical features, pathophysiology and treatment strategies. Cancer Treat Rev.

[5] Svensson E, Christiansen CF, Ulrichsen SP, Rørth MR, Sørensen HT (2017). Survival after bone metastasis by primary cancer type: a Danish population-based cohort study. BMJ Open.

[6] Guise TA, Mohammad KS, Clines G, Stebbins EG, Wong DH, Higgins LS (2006). Basic mechanisms responsible for osteolytic and osteoblastic bone metastases. Clin Cancer Res.

[7] Chen YC, Sosnoski DM, Mastro AM (2010). Breast cancer metastasis to the bone: mechanisms of bone loss. Breast Cancer Res.

[8] Hesse E. Taipaleenmäki H. MicroRNAs in Bone Metastasis. Curr Osteoporos Rep. 2019;17:122–8. 10.1007/s11914-019-00510-4 30905007

[9] Taipaleenmäki H, Browne G, Akech J, Zustin J, van Wijnen AJ, Stein JL (2015). Targeting of Runx2 by miRNA-135 and miRNA-203 impairs progression of breast cancer and metastatic bone disease. Cancer Res.

[10] D’Oronzo S, Coleman R, Brown J, Silvestris F (2019). Metastatic bone disease: pathogenesis and therapeutic options: up-date on bone metastasis management. J Bone Oncol.

[11] Schott AF, Barlow WE, Van Poznak CH, Hayes DF, Moinpour CM, Lew DL (2016). Phase II studies of two different schedules of dasatinib in bone metastasis predominant metastatic breast cancer: SWOG S0622. Breast Cancer Res Treat.

[12] Haider MT, Taipaleenmäki H (2018). Targeting the metastatic bone microenvironment by microRNAs. Front Endocrinol (Lausanne).

[13] Kaplan RN, Psaila B, Lyden D (2006). Bone marrow cells in the ‘pre-metastatic niche’: within bone and beyond. Cancer Metastasis Rev.

[14] Ren G, Esposito M, Kang Y (2015). Bone metastasis and the metastatic niche. J Mol Med (Berl).

[15] Templeton ZS, Lie W-R, Wang W, Rosenberg-Hasson Y, Alluri RV, Tamaresis JS (2015). Breast cancer cell colonization of the human bone marrow adipose tissue niche. Neoplasia (New York, NY).

[16] Price TT, Burness ML, Sivan A, Warner MJ, Cheng R, Lee CH (2016). Dormant breast cancer micrometastases reside in specific bone marrow niches that regulate their transit to and from bone. Sci Transl Med.

[17] Shiozawa Y, Pedersen EA, Havens AM, Jung Y, Mishra A, Joseph J (2011). Human prostate cancer metastases target the hematopoietic stem cell niche to establish footholds in mouse bone marrow. J Clin Invest.

[18] Psaila B, Lyden D, Roberts I (2012). Megakaryocytes, malignancy and bone marrow vascular niches. J Thromb Haemost.

[19] Wang H, Yu C, Gao X, Welte T, Muscarella AM, Tian L (2015). The osteogenic niche promotes early-stage bone colonization of disseminated breast cancer cells. Cancer Cell.

[20] Ghajar CM, Peinado H, Mori H, Matei IR, Evason KJ, Brazier H (2013). The perivascular niche regulates breast tumour dormancy. Nat Cell Biol.

[21] Calvi LM (2006). Osteoblastic activation in the hematopoietic stem cell niche. Ann N Y Acad Sci.

[22] Calvi LM, Adams GB, Weibrecht KW, Weber JM, Olson DP, Knight MC (2003). Osteoblastic cells regulate the haematopoietic stem cell niche. Nature.

[23] Zhang J, Niu C, Ye L, Huang H, He X, Tong W-G (2003). Identification of the haematopoietic stem cell niche and control of the niche size. Nature.

[24] Taichman RS (2005). Blood and bone: two tissues whose fates are intertwined to create the hematopoietic stem-cell niche. Blood.

[25] Adams GB, Chabner KT, Alley IR, Olson DP, Szczepiorkowski ZM, Poznansky MC (2006). Stem cell engraftment at the endosteal niche is specified by the calcium-sensing receptor. Nature.

[26] Lawson MA, McDonald MM, Kovacic N, Hua Khoo W, Terry RL, Down J, Kaplan W, Paton-Hough J, Fellows C, Pettitt JA, Neil Dear T, Van Valckenborgh E, Baldock PA, Rogers MJ, Eaton CL, Vanderkerken K, Pettit AR, Quinn JM, Zannettino AC, Phan TG, Croucher PI. Osteoclasts control reactivation of dormant myeloma cells by remodelling the endosteal niche. Nat Commun. 2015;6:8983. 10.1038/ncomms9983 PMC4686867 26632274

[27] Wang N, Docherty FE, Brown HK, Reeves KJ, Fowles AC, Ottewell PD (2014). Prostate cancer cells preferentially home to osteoblast-rich areas in the early stages of bone metastasis: evidence from in vivo models. J Bone Miner Res.

[28] Zalucha JL, Jung Y, Joseph J, Wang J, Berry JE, Shiozawa Y, Taichman RS. The Role of Osteoclasts in Early Dissemination of Prostate Cancer Tumor Cells. J Cancer Stem Cell Res. 2015;3:e1005. 10.14343/jcscr.2015.3e1005 PMC4469294 26097863

[29] Yeh BW, Wu WJ, Li WM, Li CC, Huang CN, Kang WY (2012). Overexpression of TG-interacting factor is associated with worse prognosis in upper urinary tract urothelial carcinoma. Am J Pathol.

[30] Wang JL, Qi Z, Li YH, Zhao HM, Chen YG, Fu W (2017). TGFβ induced factor homeobox 1 promotes colorectal cancer development through activating Wnt/β-catenin signaling. Oncotarget.

[31] Zhang M-Z, Ferrigno O, Wang Z, Ohnishi M, Prunier C, Levy L (2015). TGIF governs a feed-forward network that empowers Wnt signaling to drive mammary tumorigenesis. Cancer Cell.

[32] Saito H, Gasser A, Bolamperti S, Maeda M, Matthies L, Jähn K (2019). TG-interacting factor 1 (Tgif1)-deficiency attenuates bone remodeling and blunts the anabolic response to parathyroid hormone. Nat Commun.

[33] Shen J, Walsh CA (2005). Targeted disruption of Tgif, the mouse ortholog of a human holoprosencephaly gene, does not result in holoprosencephaly in mice. Mol Cell Biol.

[34] Allocca G, Kusumbe AP, Ramasamy SK, Wang N (2016). Confocal/two-photon microscopy in studying colonisation of cancer cells in bone using xenograft mouse models. Bonekey Rep.

[35] Kusumbe AP, Ramasamy SK, Adams RH (2014). Coupling of angiogenesis and osteogenesis by a specific vessel subtype in bone. Nature.

[36] Ubellacker JM, Baryawno N, Severe N, DeCristo MJ, Sceneay J, Hutchinson JN (2018). Modulating bone marrow hematopoietic lineage potential to prevent bone metastasis in breast cancer. Cancer Res.

[37] Dempster DW, Compston JE, Drezner MK, Glorieux FH, Kanis JA, Malluche H (2013). Standardized nomenclature, symbols, and units for bone histomorphometry: a 2012 update of the report of the ASBMR Histomorphometry Nomenclature Committee. J Bone Miner Res.

[38] Haider M-T, Holen I, Dear TN, Hunter K, Brown HK (2014). Modifying the osteoblastic niche with zoledronic acid in vivo—potential implications for breast cancer bone metastasis. Bone.

[39] Stewart AF, Wu T, Goumas D, Burtis WJ, Broadus AE (1987). N-terminal amino acid sequence of two novel tumor-derived adenylate cyclase-stimulating proteins: identification of parathyroid hormone-like and parathyroid hormone-unlike domains. Biochem Biophys Res Commun.

[40] Suva LJ, Winslow GA, Wettenhall RE, Hammonds RG, Moseley JM, Diefenbach-Jagger H (1987). A parathyroid hormone-related protein implicated in malignant hypercalcemia: cloning and expression. Science..

[41] Jüppner H (1995). Functional properties of the PTH/PTHrP receptor. Bone.

[42] Yong LK, Lai S, Liang Z, Poteet E, Chen F, van Buren G (2016). Overexpression of Semaphorin-3E enhances pancreatic cancer cell growth and associates with poor patient survival. Oncotarget.

[43] Maejima R, Tamai K, Shiroki T, Yokoyama M, Shibuya R, Nakamura M (2016). Enhanced expression of semaphorin 3E is involved in the gastric cancer development. Int J Oncol.

[44] Malik MF, Satherley LK, Davies EL, Ye L, Jiang WG (2016). Expression of Semaphorin 3C in breast cancer and its impact on adhesion and invasion of breast cancer cells. Anticancer Res.

[45] van Zijl F, Krupitza G, Mikulits W (2011). Initial steps of metastasis: cell invasion and endothelial transmigration. Mutat Res.

[46] Vallet S, Bashari MH, Fan FJ, Malvestiti S, Schneeweiss A, Wuchter P (2016). Pre-osteoblasts stimulate migration of breast cancer cells via the HGF/MET pathway. PLoS One.

[47] Xiang G, Yi Y, Weiwei H, Weiming W (2015). TGIF1 promoted the growth and migration of cancer cells in nonsmall cell lung cancer. Tumour Biol.

[48] Wang Y, Li L, Wang H, Li J, Yang H. Silencing TGIF suppresses migration, invasion and metastasis of MDA‑MB‑231 human breast cancer cells. Oncol Rep. 2018;39:802–8. 10.3892/or.2017.6133 29207164

[49] Wang Y, Shi L, Li J, Li L, Wang H, Yang H (2019). Long-term cadmium exposure promoted breast cancer cell migration and invasion by up-regulating TGIF. Ecotoxicol Environ Saf.

[50] Kumanogoh A, Kikutani H (2013). Immunological functions of the neuropilins and plexins as receptors for semaphorins. Nat Rev Immunol.

[51] Movassagh H, Shan L, Halayko AJ, Roth M, Tamm M, Chakir J (2014). Neuronal chemorepellent Semaphorin 3E inhibits human airway smooth muscle cell proliferation and migration. J Allergy Clin Immunol.

[52] Bribián A, Nocentini S, Llorens F, Gil V, Mire E, Reginensi D (2014). Sema3E/PlexinD1 regulates the migration of hem-derived Cajal-Retzius cells in developing cerebral cortex. Nat Commun.

[53] Movassagh H, Saati A, Nandagopal S, Mohammed A, Tatari N, Shan L (2017). Chemorepellent Semaphorin 3E negatively regulates neutrophil migration in vitro and in vivo. J Immunol.

[54] Wu JH, Li Y, Zhou YF, Haslam J, Elvis ON, Mao L (2017). Semaphorin-3E attenuates neointimal formation via suppressing VSMCs migration and proliferation. Cardiovasc Res.

[55] Hughes A, Kleine-Albers J, Helfrich MH, Ralston SH, Rogers MJ (2012). A class III semaphorin (Sema3e) inhibits mouse osteoblast migration and decreases osteoclast formation in vitro. Calcif Tissue Int.

[56] Ueda Y, Kondo N, Ozawa M, Yasuda K, Tomiyama T, Kinashi T (2016). Sema3e/Plexin D1 modulates immunological synapse and migration of thymocytes by Rap1 inhibition. J Immunol.

[57] Alamri A, Rahman R, Zhang M, Gounni AS, Kung SKP (2018). Semaphorin-3E produced by immature dendritic cells regulates activated natural killer cells migration. Front Immunol.

[58] Croucher PI, McDonald MM, Martin TJ (2016). Bone metastasis: the importance of the neighbourhood. Nat Rev Cancer.

[59] Butler JM, Kobayashi H, Rafii S (2010). Instructive role of the vascular niche in promoting tumour growth and tissue repair by angiocrine factors. Nat Rev Cancer.

[60] Gunatillake T, Yong HE, Dunk CE, Keogh RJ, Borg AJ, Cartwright JE (2016). Homeobox gene TGIF-1 is increased in placental endothelial cells of human fetal growth restriction. Reproduction.

