# Supplementary material for: Breast cancer bone metastases are attenuated in a Tgif1-deficient bone microenvironment
Source: Breast Cancer Res. 2020 Apr 9;22:34. doi: 10.1186/s13058-020-01269-8 (PMC7146874; doi:10.1186/s13058-020-01269-8)
Supplement: Supplementary file 1 — Additional file 1: Supplemental Figure 1. Illustration of the histological analysis. [file 13058_2020_1269_MOESM1_ESM.pdf]

# Supplemental Figure 1

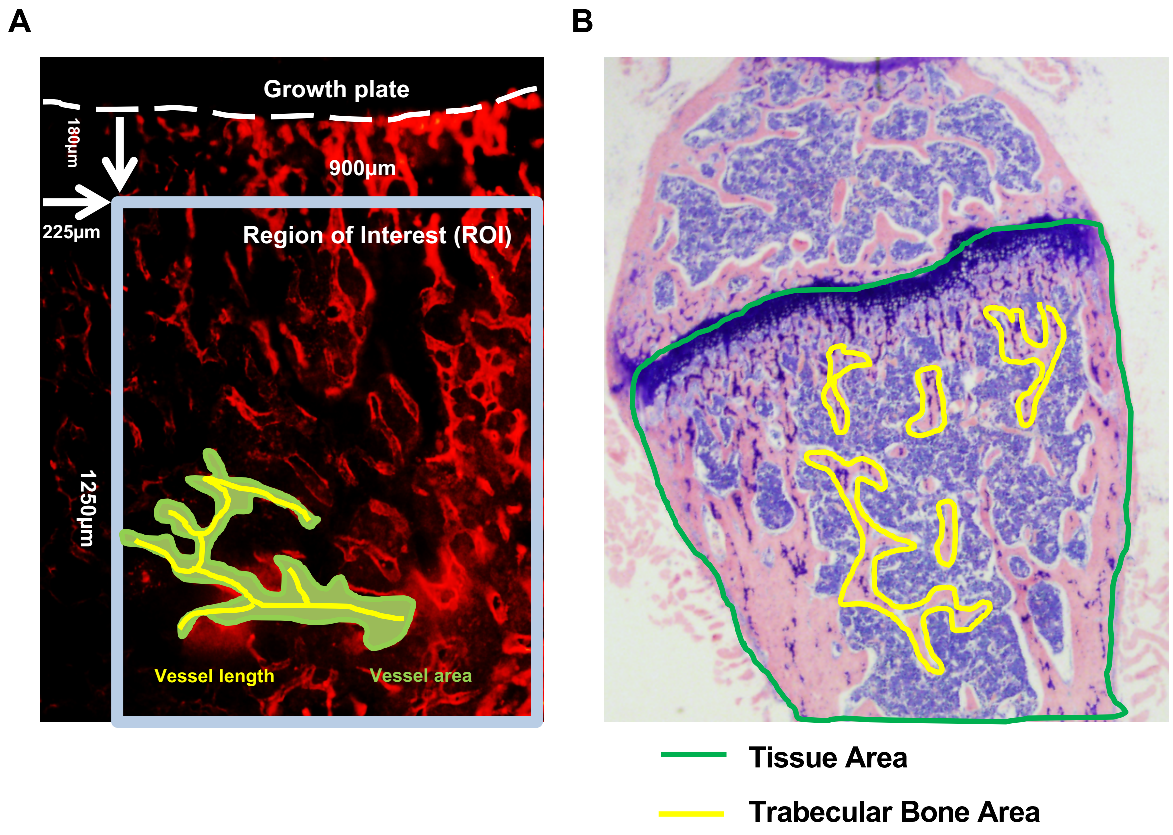


**Supplemental Figure 1. Illustration of the histological analysis. A**, Analysis of the bone marrow vasculature was performed on 30 µm thick gelatin-embedded sections of tibiae from mice that were sacrificed 5 days after 4T1-GFP tumor cell injection. Only sections without tumor cells were included in the analysis. Sections were stained with anti-Endomucin antibody and 3-4 non-serial sections per mouse were quantified. The vessel length (yellow line), vessel area (green) and number of the Endomucin-positive vessels were quantified by interactive drawing using the Osteomeasure system. **B**, Trabecular bone area per tissue area was quantified on 5µm thick sections of femurs after Giemsa staining.
