# Supplementary material for: Breast cancer bone metastases are attenuated in a Tgif1-deficient bone microenvironment
Source: Breast Cancer Res. 2020 Apr 9;22:34. doi: 10.1186/s13058-020-01269-8 (PMC7146874; doi:10.1186/s13058-020-01269-8)
Supplement: Supplementary file 2 — Additional file 2: Supplemental Figure 2. PTHrP and cancer conditioned medium increase Tgif1 expression in osteoblasts in vitro. [file 13058_2020_1269_MOESM2_ESM.pdf]

Supplemental Figure 2

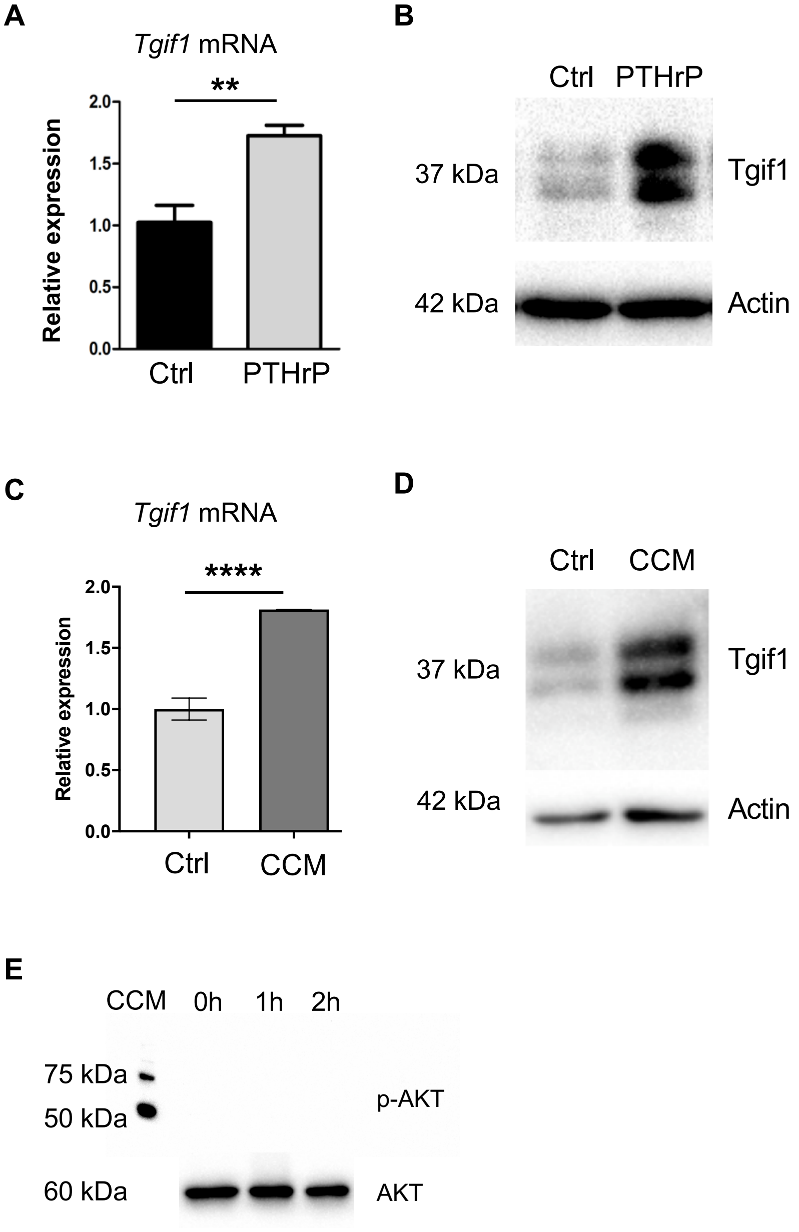

Supplemental Figure 2. PTHrP and cancer conditioned medium increase Tgif1 expression in osteoblasts *in vitro.* **A**, Tgif1 mRNA expression in osteoblasts upon stimulation with PTHrP compared to vehicle control (Ctrl). **B**, Immunoblot of Tgif1 protein expression in osteoblasts stimulated with PTHrP. Immunoblot of actin was used as loading control. **C**, Tgif1 mRNA expression in osteoblasts after 6 hours stimulation with control medium (Ctrl) or medium conditioned by breast cancer cells (CCM). **D**, Immunoblot of Tgif1 protein expression in osteoblasts upon stimulation with CCM. Immunoblot of actin was used as loading control. **E**, Immunoblot of phosphorylated AKT (p-AKT) and total AKT in osteoblasts upon stimulation with CCM. Data are presented as mean±SEM. Two-tailed Student´s t-test was used to compare two groups, **<0.01, ****<0.0001.
