# Supplementary material for: Breast cancer bone metastases are attenuated in a Tgif1-deficient bone microenvironment
Source: Breast Cancer Res. 2020 Apr 9;22:34. doi: 10.1186/s13058-020-01269-8 (PMC7146874; doi:10.1186/s13058-020-01269-8)
Supplement: Supplementary file 3 — Additional file 3: Supplemental Figure 3. Analysis of tumor cell distribution and bone turnover markers in cancer-bearing mice. [file 13058_2020_1269_MOESM3_ESM.pdf]

Supplemental Figure 3

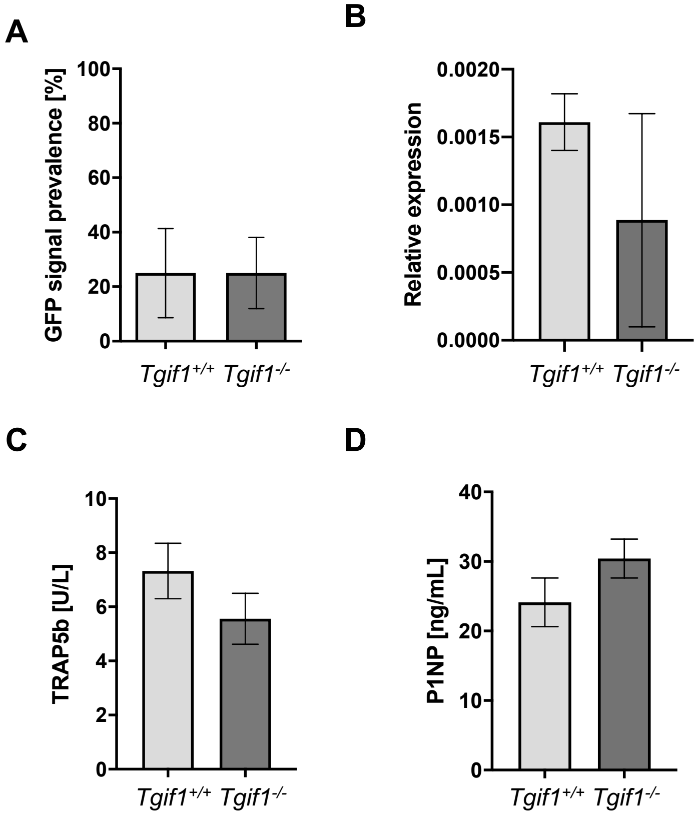

Supplemental Figure 3. Analysis of tumor cell distribution and bone turnover markers in cancer-bearing mice. A, Presence of breast cancer cells in the lungs of *Tgif1*$^{+/+}$ and *Tgif1*$^{-/-}$ mice 5 days after intracardiac injection of 4T1-GFP cells. **B**, GFP mRNA expression in the lungs of *Tgif1*$^{+/+}$ and *Tgif1*$^{-/-}$ mice (n=2-3/group). GFP expression was normalized to the expression of the house keeping gene B2M. **C** and **D**, Serum concentration of TRAP5b and P1NP in *Tgif1*$^{+/+}$ and *Tgif1*$^{-/-}$ (n=6-8 mice/group). Data are presented as mean±SEM. Two-tailed Student´s t-test was used to compare two groups.
