# Supplementary material for: Breast cancer bone metastases are attenuated in a Tgif1-deficient bone microenvironment
Source: Breast Cancer Res. 2020 Apr 9;22:34. doi: 10.1186/s13058-020-01269-8 (PMC7146874; doi:10.1186/s13058-020-01269-8)
Supplement: Supplementary file 4 — Additional file 4: Supplemental Figure 4. Analysis of the bone phenotype of Tgif1+/+ and Tgif1-/- mice. [file 13058_2020_1269_MOESM4_ESM.pdf]

Supplemental Figure 4

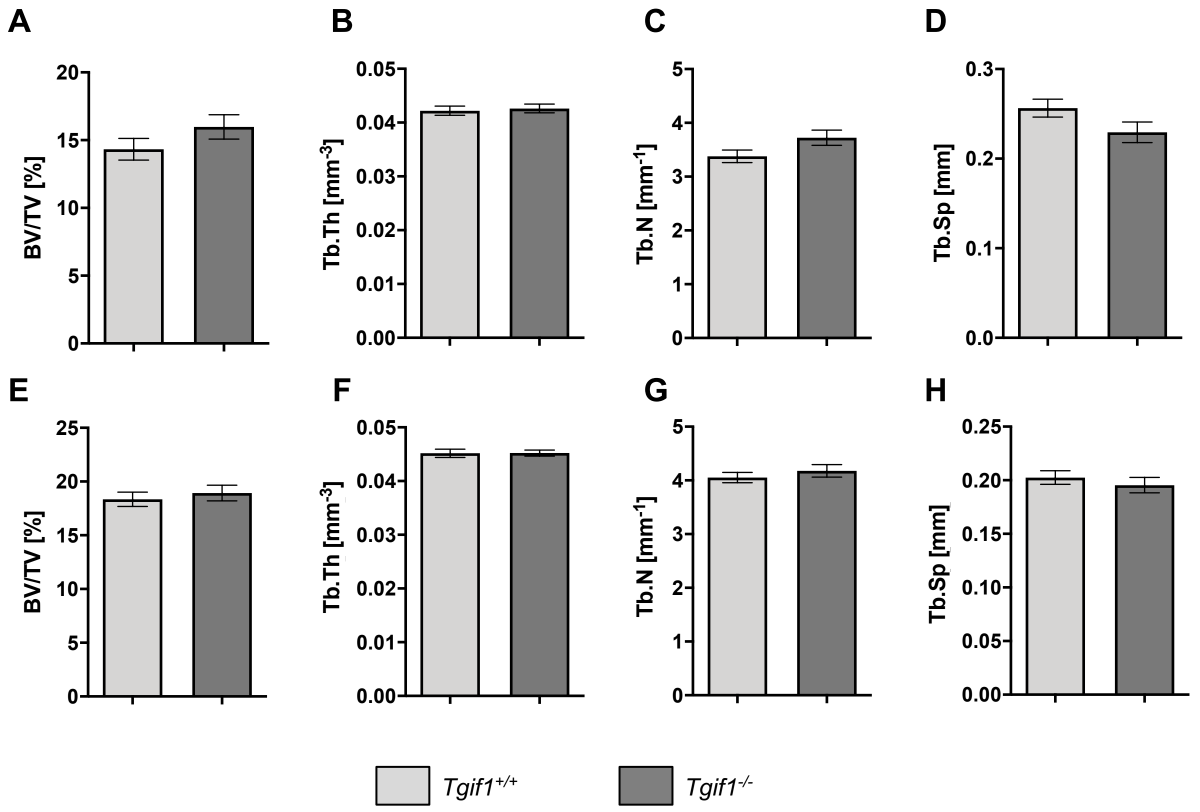

Supplemental Figure 4. Analysis of the bone phenotype of *Tgif1*$^{+/+}$ and *Tgif1*$^{-/-}$ mice. A-D, Micro-computed tomography (µCT) analysis of the trabecular bone mass (BV/TV, bone volume/tissue volume) (**A**), trabecular number (Tb.N) (**B**), trabecular thickness (Tb.Th) **(C)** and trabecular separation (Tb.Sp) **(D)** of right tibiae. **E - H**, µCT analysis of tibiae from mice five days after tumor cell injection (n≥8). For abbreviations, see **A-D**. Data are presented as mean±SEM. Two-tailed Student´s t-test was used to compare two groups.
